# High Plasma Angiopoietin-2 Levels Predict the Need to Initiate Dialysis within Two Years in Patients with Chronic Kidney Disease

**DOI:** 10.3390/ijms241210036

**Published:** 2023-06-12

**Authors:** Anna Szymczak, Mariusz Kusztal, Tomasz Gołębiowski, Krzysztof Letachowicz, Anna Goździk, Katarzyna Kościelska-Kasprzak, Andrzej Tukiendorf, Magdalena Krajewska

**Affiliations:** 1 Department of Nephrology and Transplantation Medicine, Wroclaw Medical University, 50-556 Wroclaw, Poland; 2 Institute of Cardiology, Wroclaw Medical University, 50-556 Wroclaw, Poland; 3 Institute of Health Sciences, University of Opole, 45-060 Opole, Poland

**Keywords:** angiopoietin-2, endothelial activation, overhydration, chronic kidney disease

## Abstract

Volume status, congestion, endothelial activation, and injury all play roles in glomerular filtration rate (GFR) decline. In this study, we aimed to determine whether the plasma endothelial and overhydration markers could serve as independent predictors for dialysis initiation in patients with chronic kidney disease (CKD) 3b-5 (GFR < 45 mL/min/1.72 m^2^) and preserved ejection fraction. A prospective, observational study in a single academic center was conducted from March 2019 to March 2022. Plasma levels of angiopoietin (Ang)-2, Vascular Endothelial Growth Factor-C (VEGF-C), Vascular Cell Adhesion Molecule-1 (VCAM-1), Copeptin (CPP), beta-trace protein (BTP), brain natriuretic peptide (BNP), and cardiac troponin I (cTnI) were all measured. Lung ultrasound (US) B-lines, bioimpedance, and echocardiography with global longitudinal strain (GLS) were recorded. The study outcome was the initiation of chronic dialysis (renal replacement therapy) during 24 months of follow-up. A total of 105 consecutive patients with a mean eGFR of 21.3 mL/min/1.73 m were recruited and finally analyzed. A positive correlation between Ang-2 and VCAM-1 and BTP was observed. Ang-2 correlated positively with BNP, cTnI, sCr, E/e′, and the extracellular water (ECW)/intracellular water (ICW) ratio (ECW/ICW). After 24 months, a deterioration in renal function was observed in 47 patients (58%). In multivariate regression analysis, both VCAM-1 and Ang-2 showed independent influences on risk of renal replacement therapy initiation. In a Kaplan-Meier analysis, 72% of patients with Ang-2 concentrations below the median (3.15 ng/mL) survived without dialysis for two years. Such an impact was not observed for GFR, VCAM, CCP, VEGF-C, or BTP. Endothelial activation, quantified by plasma levels of Ang-2, may play a key role in GFR decline and the need for dialysis initiation in patients with CKD 3b, 4, and 5.

## 1. Introduction

Chronic kidney disease (CKD) affects about 13.4% (11.7–15.1%) of the population worldwide. Traditional risk factors for CKD development and progression, such as diabetes mellitus (DM), hypertension, and obesity, are already well-constituted [1]. To date, the diagnosis and prognosis are based on serum creatinine, albuminuria, and proteinuria, which constitute established biomarkers. Kidney biopsies help to predict the course of the disease, but are invasive procedures [2]. The clinicians’ effort currently concentrates on finding nontraditional CKD risk factors, which, when well-described, can become good novel CKD progression markers, providing vital diagnostic and prognostic information.

The potential to become such a microvascular biomarker is present in several molecules. Among those analyzed in this study, angiopoietin-2 (Ang-2), a modulator of endothelial dysfunction [3], earns a special significance. Angiopoietins are glycoproteins required for the formation, maturation, and regulation of vessels in both vascular and lymphatic mammalian systems. Angiopoietin-2 acts through tyrosine kinase with the immunoglobulin and epidermal growth factor homology domains 2 (TIE2). TIE2 is expressed mainly in endothelial cells and plays a composite role in the development of vessels, angiogenesis, and inflammation [3,4]. Both Angiopoietin-2 and the related Angiopoietin-1 (Ang-1) are ligands for TIE2 [3,5]. The Ang-1/TIE2 structure promotes vascular stability and the anti-inflammatory homeostatic state. It is mediated, among others, by the inhibition of the endothelial growth factor (VEGF) -induced expression of vascular cell-adhesion molecule 1 (VCAM1) and intercellular adhesion molecule 1 (ICAM1). VCAM1, expressed predominantly in endothelial cells, plays an important role in vascular endothelium homeostasis [6]. Liberated from Weibel-Palade bodies in endothelial cells (EC), Ang-2, despite of being a TIE2 agonist, antagonizes Ang1 activity on the TIE2 receptor [7]. Context-dependent Ang-2 signaling, by sensitizing vessels to proangiogenic cytokines in the presence of VEGF-C, promotes sprouting angiogenesis by facilitating the survival, proliferation, and migration of EC. VEGF-C has been studied as a candidate biomarker of hypervolemia in chronic kidney disease (CKD) patients. Both Angiopoietin-2 and VCAM induce vascular permeability by pericyte loss, which leads to the disruption of the vascular barrier integrity [3,8,9]. The Ang-2/Ang-1 ratio could be considered as a biomarker of endothelial activation [10]. On a daily basis, the ratio is low due to the modest constitutive Ang-2 expression in a stable state [11]. Hypoxia, hyperglycemia, and inflammation, by increasing Ang2 expression, promote endothelial activation [12,13]. Oscillatory shear stress, in contrast to downregulating Ang-2 expression laminar flow, also increases the Ang-2/Ang-1 [12]. Such states promote vascular leakage and sprouting angiogenesis [14]. Although Angiopoietin-2 is vital to the physiological state, it is involved in many pathological conditions. An increased level of Ang-2 is observed in sepsis [11,12], acute lung injury [10], neoplasms [12,15], congestive heart failure (HF) [16], diabetes mellitus [17], thrombofibrosis [18], atherosclerosis [12], exudative retinal diseases [19], and many others. Ang-2 concentration levels in CKD patients have been examined from a variety of perspectives. They have been assessed in patients with diabetic nephropathy as an independent predictor of commencing dialysis, major adverse cardiovascular events (MACE), and all-cause mortality [18]. A synergistic effect on adverse renal outcomes in CKD patients of fluid overload and Ang-2 has been described [20]. In the general population, serum Ang-2 concentrations correlate with serum cystatin C and the estimated glomerular filtration rate (eGFR), even when hypertensive and diabetic patients with DM type 2 are excluded [21].

We aimed to assess which of the circulating biomarkers, including angiopoietin, in CKD patients with eGFR < 45 mL/min/1.73 m^2^ and a preserved ejection fraction could be predictive for renal impairment progression and initiation of renal replacement therapy.

## 2. Results

We analyzed a total of 105 patients with CKD stages G3b-5 (mean eGFR 21.3 mL/min/1.73 m^2^) who met the inclusion criteria. A total of 35 patients were in stage 3b, 34 patients were in CKD 4, and 36 were in stage 5. The mean age at enrollment was 52.6 (±14.8) years, and 68 (64.8%) of the patients were male. Of all participants, 79% were hypertensive and 24% were diabetic mellitus. There were 22 patients with heart failure enrolled in the study. Ten of them were in the dialysis group. Regarding the etiology of CKD, the following causes were recorded: chronic glomerulonephritis (GN)—24; diabetes—22; hypertensive nephropathy—19; autosomal dominant polycystic kidney disease (ADPKD) -10; interstitial—8; urologic complication—6; unknown—11. However, we did not find a specific relation between circulating biomarkers and any of the above etiologies. Table 1 shows the baseline patient characteristics.

### 2.1. Correlations in the Entire Cohort

**Figure 1:**
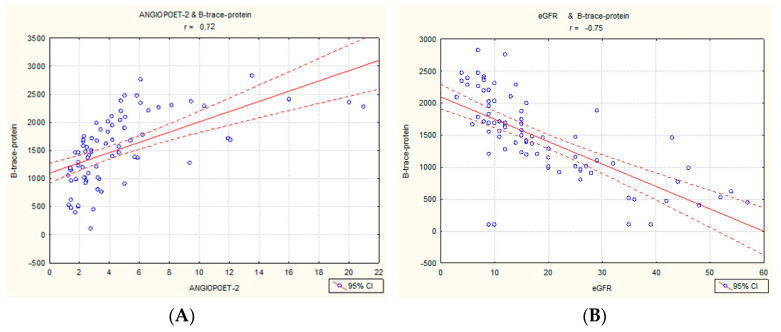
Significant strong correlation between B-trace protein and angiopoetin-2 (**A**) and between B-trace protein and eGFR (**B**).

Spearman’s and Pearson’s correlations in the entire cohort were assessed. Positive strong correlations between the concentration of angiopoetin-2 and B-trace protein (Figure 1A) as well as VCAM (r 0.56) were observed. The Ang-2 concentration also correlated positively with BNP, cTnI, serum creatinine, procalcitonin, e/e′, and ECW/ICW, and negatively with hemoglobin and eGFR (Table 2). The second-strongest correlations shown were B-trace protein (prostaglandin d synthase) with eGFR (r = −0.75, Figure 1B), serum creatinine (r = 0.74), procalcitonin (r = 0.61), and VCAM (r = 0.56). Other marked correlations were found between B-trace protein and BNP (r = 0.46) and urine osmolality (r = −0.45). The frequently used congestive marker, BNP, correlated most strongly with Ang-2, VCAM, and B-trace protein, and with the ECW/ICW index (bioimpedance) and ultrasound measures: B-lines, e/e′ (Table 2).

### 2.2. Univariate and Multivariate Regression

Table 3 displays univariate regression analysis and Table 4 shows multivariate regression on the risk of renal replacement start (yes vs. no within 24 months). Vascular cell-adhesion molecule 1 shows independent influence on the risk of renal replacement start in multivariate analysis but the effect on hazard ratio (HR) is marginal. Angiopoietin-2 shows a independent effect on the need for RRT when confront with BNP (model 2; Table 4).

Renal functions of the subjects were analyzed after 24 months. The patients were divided into three groups: with stable/ameliorated renal function (nonprogressors), deteriorated renal function (progressors), and undergoing maintenance hemodialysis. Comparative data between the groups are displayed in Table 5.

### 2.3. Ang-2, VCAM, and B-Trace Protein in Progressors vs. Nonprogressors

Over a follow-up period of 24 months, deterioration of renal function was observed in 47 patients (progressors), and 31 presented stable or ameliorated renal function. Twenty-two maintenance dialysis patients formed a control group (Table 5).

Between the two groups, progressors versus nonprogressors, we observed a difference in concentration of angiopoetin-2, but also of VCAM and B-trace protein. Moreover, differences in urine and blood osmolality and procalcitonin, as well as in traditional clinical measures such as lung ultrasound B-lines and BNP were observed (Table 5).

### 2.4. Impact of Angiopoetin-2 on Renal Survival (RRT Initiation)

**Figure 2:**
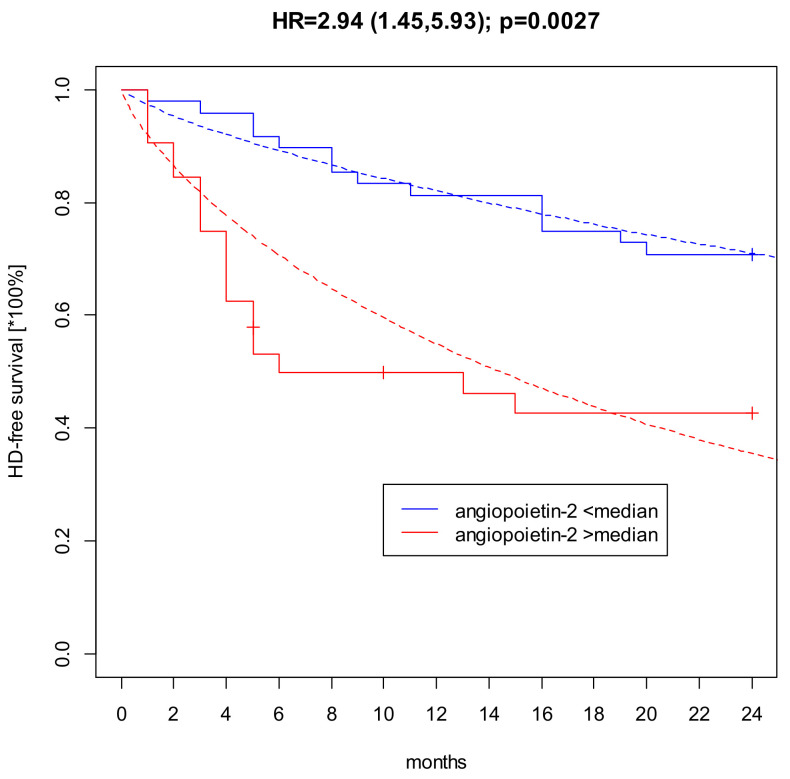
Kaplan-Meier survival curves for high vs. low Angiopoietin-2 concentrations. A high level of angiopoietin-2 (red line; >median 3.15 ng/mL) is indicative of dialysis initiation within two years. Low level of angiopoetin-2 (blue line; below median) is indicates a better prognosis.

In a Kaplan-Meier analysis, a significant impact of Ang-2 on renal survival, described as a need for RRT commencement, a hard endpoint, was found (Figure 2). Namely, 72% of patients with Ang-2 concentrations below the median (3.15 ng/mL) survived without dialysis for two years, compared to 42% of those with Ang-2 concentrations above the median (high level). Such an impact (statistical significance) was not observed for any of the biochemistry parameters (e.g., eGFR, urea, BNP), nor of the endothelial or congestion biomarkers (VCAM, Copeptin, VEGF-C, B-trace protein). Unlike eGFR, Ang-2 helps predict the need to start RRT within two consecutive years in patients with CKD 3b, 4, and 5. 

## 3. Discussion

In our study, we showed that Angiopoetin-2, unlike other examined potential biomarkers and eGFR, helps to predict the need for renal replacement therapy commencement within a two-year span. Although the concentration of B-trace protein and VEGF-C also differed significantly in the progressors and nonprogressors groups, they did not show such a predictive value.

In the angiopoietin family, Angiopoietin-1 (Ang-1) and Angiopoietin-2 (Ang-2) are crucial. Ang-1 maintains vascular quiescence, prevents vessel leakage, and promotes endothelial cell survival via activation of the endothelial cell receptor Tie-2. Ang-2 competitively inhibits the effects of Ang-1 on Tie-2, leading to vessel instability and eventual capillary rarefaction. Ang-2 plays a role in many diseases related to vascular permeability [14]. 

Regarding the association of Ang-2 with adverse renal outcomes, our study is consistent with results obtained by Tsai et al. [22], who discovered the association between Ang-2 and composite renal outcomes, either doubling serum creatinine or commencing dialysis. Patients with Ang-2 concentration in quartile 3 or more had a 1.7- to 3-fold increase in commencing dialysis risk or rapid renal function decline, and a more rapid decrease in eGFR over time showed in the mixed-effect model. Four years later, Tsai et al. [23] concentrated on diabetes mellitus CKD patients and found that in this group of patients, Ang-2 was significantly associated with the risk of meeting not only hard renal endpoints, that is, dialysis commencement and rapid eGFR decline, but also MACEs and all-cause mortality. 

As chronic kidney disease predisposes one to cardiovascular disease [24], and Ang-2 has been associated with cardiovascular disease [25], Tsai et al. [26] aimed to analyze the association of serum markers of angiogenesis with subclinical measures of cardiovascular function and structure in patients with eGFR < 60 mL/min/1.73 m^2^. In this study, Ang-2, Ang-1, and VEGF levels, as well as echocardiography parameters, were all measured. Ang-2 was significantly associated with left ventricle mass index and hypertrophy. Although in pathophysiology VEGF-A plays an important role in Ang-2 mediated signaling modulation, the study did not reveal a significant correlation with cardio vasculature. The same team (Tsai et al. [27]) conducted a study on predialysis CKD patients in stages 3–5, in which the relationship between Ang-2 and MACE and all-cause mortality was assessed. It turned out that patients in the quintile of the highest Ang-2 concentration had double the risk for MACE or all-cause mortality after adjustment of associated risk factors over the follow-up period of three years.

On the basis of a model of CKD development in mice, the first therapeutic strategy directly addressing renal endothelium via Ang-2 inhibition as a CKD therapy was established. Chang et al. [28] showed that Ang-2 inhibition, obtained by administration of recombinant L1–10 (a Fc-fusion peptide able to block Ang-2 binding to receptor Tie-2) or native Ang-1 overexpression, can reduce kidney fibrosis. This is performed via debilitation of inflammatory processes and apoptosis in endothelia. The pathogenesis is explained by the observation that Ang-2 enhances endothelial macrophage infiltration and triggers apoptosis in fibrosing kidneys. Moreover, the assumption that higher Ang-2 and Ang-2/Ang-1 ratios are positively associated with end stage kidney disease risk in CKD patients was verified. Since an effective treatment that could stop the progression of CKD is still unknown, this finding could help to provide new therapeutic options. 

In a small study, Bontekoe et al. [29] hypothesized that Ang-2, as a vascular remodeling, endothelial instability, and inflammation biomarker, is a common denominator of atrial fibrillation (AF) and Stage 5 CKD on hemodialysis (CKD5-HD). Ang-2 levels were found to be higher in the group with coexisting CKD5-HD and AF than in CKD5-HD alone and AF alone, which suggests an additive effect of Ang-2 with concomitance of AF and CKD5-HD.

According to our study, there is a moderate link between Ang-2 and the E/e′ ratio, which is a measure of the LV filling pressure [30]. Diastolic dysfunction with E/e′ > 15 is not a rare finding in CKD patients, and CV risk increases as renal function declines [31]. Kim et al. [32] analyzed 84 patients to conclude that there is an association between E/e′ ratio and fluid overload in patients with CKD in the predialysis period. Patients would benefit from the correction of overhydration, as it would decrease CV risk; therefore, the authors recommended the regular monitoring of fluid status. According to the paper by Yang et al. [33], B-lines measured during LUS positively correlate with E/e′ in patients, especially those with preserved ejection fraction heart failure (HFpEF). The authors observed that B-lines ≥25 in HFpEF patients allow for the detection of E/e′ ≥14 with 92% sensitivity and 83% specificity. All patients enrolled in our study showed preserved EF (≥50%). We found a moderate correlation between B-lines and Ang-2. 

The evaluation of renal function is mostly focused on calculating GFR using creatinine calculations. Lipocalin-type prostaglandin D2 synthase (prostaglandin H2-D-isomerase), also known as beta-trace protein (BTP), is a low molecular weight (LMW) protein. It catalyzes the conversion of prostaglandin H2 (PGH2) to prostaglandin D2 (PGD2). BTP—as LMW protein shares with Cystatin-C (CysC) biochemical features exceptionally useful in GFR estimation. Beta-trace protein (BTP; prostaglandin D- Synthase) has been nominated in the last few years as a potential new biomarker of renal function decline [34]. BTP is frequently compared to GFR as an alternative marker of renal failure due to minimal extrarenal elimination and no tubular secretion. In our study, we found strong correlations between BTP and eGFR, as well as between BTP and Ang-2. Compared to the traditional creatinine-based equations, BTP-based equations improved the risk associations and quite improved the prediction for end stage kidney disease and mortality. Moreover, the best performance was found using an eGFR equation that included a combination of creatinine and BTP [34].

It is highly likely that the combination of Ang-1 and Ang-2 tests will greatly enhance the risk stratification for CKD development and dialysis commencement. In a recent prospective multicenter investigation, the prognostic value of angiopoietins (Ang-1:Ang-2 ratio) for future renal disease in patients with acute kidney injuries was determined [35]. We hypothesize that angiopoietins may explain the course of renal disease in both acute kidney disease and CKD via a similar mechanism.

The limitation of our study was lack of evaluation of the interaction between the therapeutic intervention and the biomarker. All patients were ambulatory, and we considered them as clinically compensated, receiving the standard of care medication (antihypertensives, diuretics, etc.). Furthermore, only half of the study patients had urine albumin creatinine ratio (UACR) results (properly taken morning sample) available, and all of them showed positive results (UACR >  30 mg/g). Nevertheless, ranges were similar in the majority of the patients (no patients showed nephrotic or nephritic syndrome), and in this study we focused on CKD patients who would start dialysis in near future. Future research should analyze how currently used medication changes the levels of angiopoietins. 

## 4. Materials and Methods

### 4.1. Participants

The observational study was conducted at the University Hospital Wroclaw between March 2019 and March 2022. The study group included 105 patients with chronic kidney disease in stages G4–5, including kidney transplantation recipients. Patients undergoing hemodialysis were used as a control group. The decision to start dialysis was based on GFR < 15 mL/min and at least one of the uremic symptoms (dyspnea, weight gain with diuresis reduction < 800 mL/24 h, uncontrolled anemia, uncontrolled hypertension, lack of appetite, fetor uremicus, pruritus, lack of energy). CKD was staged according to to Kidney Disease Improving Global Outcomes (KDIGO 2012) GFR categories and the eGFR was calculated using the simplified four-variable Modification of Diet in Renal Disease (MDRD) Study formula [36]. The inclusion criteria were an eGFR of less than 45 mL/min/1.73 m^2^, left ventricle ejection fraction (LVEF) >45%, age above 18 years, and informed consent. The exclusion criteria were liver cirrhosis, an active malignancy, severe lung disease, LVEF < 45%, and NYHA III or IV. 

Diagnosis criteria for heart failure were based on echocardiography (EF < 50% and/or left ventricular end-diastole diameter greater than 5.5 cm) and/or BNP levels: over 500 pg/mL for patients with CKD stages 3b; over 800 pg/mL for stage 5. In the dialysis group there were six patients with HF with preserved EF (HFpEF), EF > 50%; four patients had HF with mildly reduced EF (HFmrEF), EF 45–49%.

One patient died due to COVID-19 infection, and four others were lost in the follow-up period. 

### 4.2. Study Design

The patients were assessed at the enrollment, which took place between March 2019 and November 2020. Demographic and clinical data included age, body mass index (BMI), presence of diabetes mellitus, and hypertension (HTN). The follow-up data were collected based on medical documentation and telephone contact with patients 24 months after the enrollment. The observation ended in March 2022. In each participant we performed a lung ultrasound (LUS) examination with a B-lines (ultrasound B-lines) assessment, echocardiography with global longitudinal strain evaluation, a pulse wave velocity measurement, bioelectrical impedance analysis (BIA), and a six-minute walk test (6-MWT) for evaluating physical functional capacity. All the exams and measurements, despite echocardiography, which was performed by a cardiologist, were performed by the same nephrologist. Blood and urine samples were collected for standard laboratory tests (serum creatinine, eGFR, urea, and uric acid; C-reactive protein and procalcitonin; total protein and albumin; hemoglobin, brain natriuretic peptide, cardiac troponin I, and sodium; osmolality of serum and urine) and for potential novel markers of renal insufficiency. An accredited local laboratory performed measures of concentration using Enzyme-Linked Immunosorbent Assay (ELISA) for Angiopoetin-2, Vascular Endothelial Growth Factor-C, Vascular Cell Adhesion Molecule-1 (CD106), Copeptin, and Prostaglandin D- Synthase (B-trace protein). 

#### 4.2.1. Lung Ultrasonography (B-Lines)

A standard (3.0-MHz) echocardiography probe (Samsung HS50, Suwon, Republic of Korea) was used for the chest ultrasound. The test protocol encompassed detecting extravascular lung water (LW) by visualizing B-lines, as these are the US equivalent of standard chest X-ray Kerley B-lines [37]. The examined patients were lying in a supine position. We were visualizing and counting comets in 28 intercostal spaces on both sides of the chest, that is, from the second to the fifth (on the left side to the fourth) intercostal spaces at the parasternal, midclavicular, anterior, and middle axillary lines. Comets are the hyperechoic reverberation artefacts formed between the pleura and subpleural thickened in the presence of excessive LW-interlobular septa [38]. On the ultrasound screen they are visible as coherent US bundles running from the transducer to the screen edge. It is already known that pulmonary congestion in patients with HF, or patients who are chronically overhydrated due to end-stage renal insufficiency, is reflected in the presence of comets [39,40].

#### 4.2.2. Echocardiography with GLS

The transthoracic echocardiography examination was performed with a high-resolution ultrasound machine (Vivid E 9, GE Healthcare, Horten, Norway). On the basis of the measurements, the LV mass (LVM) was calculated and then indexed to the body surface area (BSA). For the diastolic function evaluation, we used the ratio between early mitral inflow velocity and mitral annular early diastolic velocity (E/e′) visualized in pulse-wave (PW) and tissue Doppler, respectively. Ejection fraction of the left ventricle was calculated by the Simpson biplane method. GLS was measured using a two-dimensional speckle tracking echocardiography. Three apical views were obtained, and then the average peak systolic longitudinal strain was provided by the software. 

#### 4.2.3. Bioimpedance

The bioelectrical impedance analysis was measured with a single-frequency (SF) analyzer BIA-101 (Akern, Pontassieve, Italy). The measurements were conducted according to the manufacturers’ instructions, at one frequency of 50 kHz at 0.8 mA. Every patient was examined in a supine position. The single-use dedicated electrodes were attached to one hand and ipsilateral ankle. After being acquired, the resistance and reactance values, together with other basic characteristics of the patient, were analyzed by dedicated software. Extracellular water and intracellular water were obtained, and then the ECV/ICV ratio was counted. 

#### 4.2.4. Six-Minute Walk Test

The 6-MWT is an accessible and easy-to-perform test with wide indications [41]. For the purpose of this study, we included in the examination the evaluation of cardiopulmonary and functional capacities. A stopwatch, a pulse oximeter, and a sphygmomanometer were used by the observer present. A 25-m-long section of a flat and straight corridor was used; the patients were instructed to walk back and forth, and the total walked distance was measured. Before the walk, and immediately after cessation of the walk, pulse and blood pressure were measured. Pulse was also measured after one minute of rest. 

#### 4.2.5. Quantification of Circulating Biomarkers

For VCAM-1, VEGF-C, and Angiopoietin-2 concentration measurements, the Quantikine™ ELISA (R&D Systems Inc, Minneapolis, MN, USA) solid-phase sandwich technique was used. For prostaglandin D synthase, the sandwich ELISA (BioVendor, Brno, Czech Republic) was also used. For Copeptin (CPP), a Competitive-ELISA (Novus Biologicals, Centennial, CO, USA) set was applied. After the sequence of reactions of each biomarker in 96-well polystyrene microplates precoated with proper antibodies, the color change was measured spectrophotometrically at a wavelength of 450 nm. The concentrations of each biomarker in the examined samples were then determined by comparing the optical density of the samples to the standard curve. The sensitivity for Ang-2, expressed as mean minimum detectable dose (MDD), was 8.29 pg/mL.

Based on renal function after 24 months, patients were divided into three groups: with stable/ameliorated renal function; with deteriorated renal function; and undergoing hemodialysis as a control group. 

### 4.3. Statistical Analysis

Statistical analysis was performed using Statistica ver. 13 software (StatSoft, Tulsa, OK, USA). Data in tables are expressed as the mean and median with interquartiles (IQR). The distribution was tested with the use of the Kolmogorov–Smirnov test. Differences between the two groups were assessed with the use of a t-test or median U test. Categorical variables were expressed as absolute (N) and percentage (%) values and compared using the χ^2^ test. Univariate and multivariate regression analyses were performed to identify clinical factors influencing the risk of dialysis starting. Kaplan-Meier survival analysis was used to analyze the impact of plasma biomarkers, e.g., Ang-2, on renal replacement therapy (RRT) commencement. Statistical significance was set at a two-sided *p*-value of less than 0.05.

## 5. Conclusions

In conclusion, our study demonstrated that among other new potential biomarkers, unlike GFR, elevated Angiopoetin-2 concentration shows predictive properties regarding the necessity to commence RRT in CKD patients in Stages 3b-5. Understanding of the pathomechanism underlying the link between adverse renal outcomes and increased circulation of Ang-2 can help not only due to its predictive value, but maybe also in developing future therapeutic strategies. 

## Figures and Tables

**Table 1:** Demographic clinical, biochemical, bioimpedance, and ultrasound characteristics of the patients at baseline.

Parameter	Mean	Median	25–75% [IQR]
Age (years)	52.6	52.0	42–66
Body mass index (kg/m^2^)	25.9	25.7	22.2–27.9
6-MWT (m)	407.6	420.0	360–480
Sys BP (mmHg)	144.3	144.0	127–160
Dia BP (mmHg)	87.4	85.0	80–100
Heart rate (1/min)	71.6	70.0	62–81
Biochemistry			
Serum creatinine (mg/dL)	4.3	3.8	2.3–5.8
eGFR (mL/min/1.73 m^2^)	21.9	16.0	10–28
Urea (mg/dL)	105.2	97.0	64–142
BNP (ng/mL)	376.7	103.3	37.6–287.8
cTnI (ng/dL)	17.4	8.9	4.3–18.8
Hemoglobin (mmol/L)	11.2	11.0	9.6–12.4
Sodium (mmol/L)	140.3	140.0	139–142
Urine osmolality (mOsm/kg H_2_O)	338.3	335.5	259–402
Serum osmolality (mOsm/kg H_2_O)	303.2	303.5	295–313
Uric acid (mg/dL)	7.3	7.3	6.3–8.3
Serum albumin (g/dL)	3.6	3.6	3.2–4.1
Total protein (g/dL)	6.2	6.2	5.5–6.9
C-reactive protein (mg/L)	7.7	2.4	1.1–6
Procalcitonin (ng/mL)	0.1	0.1	0.0–0.1
Ultrasound examination			
B-lines (Lung US)	10.8	7.0	3–13
LVEF (%)	61.4	65.0	60–65
GLS	−17.6	−18.0	−19.8–−14.9
Mitral E/e′	8.9	8.3	6.7–10.2
LVM index	130.7	123.0	99–156
Bioimpedance			
ECW (L)	22.5	21.7	17.1–26.1
ICW (L)	23.8	23.2	18–27.4
ECW/ICW	1.0	0.9	0.8–1.1
Biomarkers of congestion or endothelial injury/activation			
VCAM-1 (ng/mL)	1476.1	1358.0	1081.5–1759.0
ANGIOPOETIN-2 (ng/mL)	4.3	3.1	2.3–5.0
VEGF-C (ng/mL)	4.9	4.7	3.7–5.8
Copeptin (pg/mL)	893.8	574.0	342.5–1432.5
B-trace-protein (ng/mL)	1537.4	1497.0	1095–1993

6-MWT—6-min walk test; eGFR—estimated glomerular filtration rate; BNP—brain natriuretic peptide; cTnI—cardiac troponin;IQR—interquartile range; Sys BP—systolic blood pressure; Dia BP—diastolic blood pressure LUS—lung ultrasonography; LVEF—Left Ventricle Ejection Fraction; GLS—global longitudinal strains; Mitral E/e′ ratio—noninvasive measure of left ventricular filling pressure; LVM—left ventricle mass; ECW—extracellular water; ICW—intracellular water; VCAM-1—vascular cell-adhesion molecule 1; VEGF-C—vascular endothelial growth factor; B-trace-protein—prostaglandin d synthase.

**Table 2:** Correlation matrix among clinical parameters and congestion and endothelial markers.

	BMI	ECW/ ICW	6 MWT	B-Lines	e/e′	EF	GLS	VCAM-1	Ang-2	VEGF-C	CPP	B-TP
Age	0.14	0.35 *	**−0.47**	0.13	0.28	0.02	0.10	−0.10	−0.06	−0.19	**−0.33**	−0.12
BNP	−0.11	**0.46**	**−0.38**	**0.40**	**0.42**	−0.10	**0.28**	**0.50**	**0.58**	−0.12	0.00	**0.46**
cTnI	−0.16	**0.31**	**−0.35**	**0.32**	**0.45**	−0.13	**0.41**	**0.44**	**0.42**	−0.02	−0.13	0.15
Hemoglobin	0.19	**−0.48**	**0.33**	**−0.33**	−0.01	0.01	−0.01	**−0.21**	**−0.48**	**0.20**	−0.01	**−0.39**
Na	−0.12	0.28	−0.11	0.05	−0.09	0.02	−0.18	0.18	0.17	−0.11	−0.07	−0.01
U osmolality	0.18	0.06	0.20	−0.02	−0.03	**0.21**	0.06	−0.40	**−0.30**	−0.03	−0.12	**−0.45**
S osmolality	0.08	0.06	−0.11	0.11	−0.11	0.06	−0.11	0.09	**0.23**	−0.02	−0.02	**0.22**
Uric acid	0.26	0.01	0.10	−0.10	**−0.28**	0.13	**−0.35**	−0.17	**−0.18**	**−0.21**	−0.03	**−0.42**
S creatinine	−0.10	0.08	−0.09	**0.26**	−0.04	−0.11	0.03	**0.48**	**0.57**	0.03	−0.01	**0.74**
eGFR	0.20	**−0.20**	0.19	**−0.29**	−0.02	0.11	−0.04	**−0.49**	**−0.61**	0.12	−0.12	**−0.75**
urea	−0.13	**0.23**	−0.18	**0.26**	−0.09	−0.02	−0.05	**0.25**	**0.30**	−0.09	0.10	**0.23**
S albumin	0.13	**−0.35**	0.15	−0.08	−0.12	−0.15	−0.03	−0.09	0.02	−0.20	−0.12	0.19
CRP	−0.12	**0.26**	**−0.31**	**0.24**	0.17	−0.01	0.01	**0.24**	**0.34**	−0.14	−0.15	0.19
procalcitonin	−0.11	**0.24**	**−0.37**	**0.37**	0.36	−0.16	0.14	**0.58**	**0.56**	0.08	−0.18	**0.61**

* Correlation coefficient; significant correlations with *p* < 0.05 are bolded. 6-MWT—6-min walk test; Ang-2—angiopoetin2-2; eGFR—estimated glomerular filtration rate; BNP—brain natriuretic peptide; BMI- body mass index; cTnI—cardiac troponin; ECW—extracellular water; ICW—intracellular water; GLS—global longitudinal strains; e/e′ ratio—noninvasive measure of left ventricular filling pressure; EF—ejection fraction; VCAM-1—vascular cell-adhesion molecule 1; VEGF-C—vascular endothelial growth factor; CPP—copeptin; BTP (beta-trace protein)—prostaglandin d synthase; U—urine; S—serum; CRP—c-reactive protein.

**Table 3:** The univariate analyses on the risk of renal replacement start.

Univariate Regression				
Risk Factor	HR	2.5%	97.5%	*p*-Value
VCAM-1	1.0010	1.0000	1.0010	0.0017
Ang-2	1.1770	1.0380	1.3350	0.0113
B-trace-protein	1.0010	1.0010	1.0020	0.0001
VEGF-C	1.0000	0.9968	1.0001	0.4520
eGFR	0.8867	0.8477	0.9276	0.0000
BNP	1.0000	0.9998	1.0010	0.2520
Copeptin	0.9998	0.9994	1.0000	0.5280

eGFR—estimated glomerular filtration rate; BNP—brain natriuretic peptide; VCAM-1—vascular cell-adhesion molecule 1; VEGF-C—vascular endothelial growth factor; Ang-2—Angiopoetin-2

**Table 4:** The multivariate Cox analyses on the risk of renal replacement start.

Regression		Univariate				Multivariate			
Model	Risk Factor	HR	2.5%	97.5%	*p*-Value	HR	2.5%	97.5%	*p*-Value
1	VCAM-1	1.0010	1.0000	1.0010	0.0017	1.0007	1.0001	1.0010	0.0144
	Ang-2	1.1770	1.0380	1.3350	0.0113	1.1179	0.9801	1.2750	0.0968
	Copeptin	0.9998	0.9994	1.0000	0.5280	0.9999	0.9994	1.0000	0.7679
2	Ang-2	1.1770	1.0380	1.3350	0.0113	1.1790	1.0157	1.3690	0.0304
	BNP	1.0000	0.9998	1.0010	0.2520	1.0000	0.9994	1.0010	0.9582

BNP—brain natriuretic peptide; VCAM-1—vascular cell-adhesion molecule 1; Ang-2—angiopoietin 2.

**Table 5:** Comparative data between group progressors, nonprogressors, and HD maintenance.

Parameter	Group I, *n* = 31 (Nonprogressors)		Group II, *n* = 47 (Progressors)		Dialysis Group (CKD5-HD), *n* = 22		*p* < 0.05
	Median	25–75% [IQR]	Median	25–75% [IQR]	Median	25–75% [IQR]	
Age (years)	51.0	42–64	59.0	44–68	52.5	38.5–65.5	Ns
Body mass index (kg/m^2^)	24.6	22.3–27	25.7	22.3–28	25.4	21.5–30	Ns
6-MWT (m)	440.0	395–500	400.0	360–475	390.0	320–460	I vs. II, I vs. HD
Sys BP (mmHg)	144.0	125–150	140.0	127–150	149.5	127.5–170	Ns
Dia BP (mmHg)	85.0	80–94	83.0	80–93	97.0	80–100	I vs. HD, II vs. HD
Heart rate (1/min)	71.5	63–75	70.0	62–80	69.0	58–90	Ns
Serum creatinine (mg/dL)	2.3	1.5–3.2	3.8	2.4–5.2	6.7	5.4–7.6	I vs. II, I vs. HD, II vs. HD
eGFR (mL/min/1.73 m^2^)	28.0	22–46	17.0	11–27	9.0	7.5–11	I vs. II, I vs. HD, II vs. HD
24 M eGFR (mL/min/1.73 m^2^)	31.0	23–50	6.0	5–13	6.0	30–54	I vs. II, I vs. HD
Urea (mg/dL)	76.0	58–93	112.0	66–142	131.0	70–158	I vs. II, I vs. HD
BNP (ng/mL)	56.6	23.2–208	105.2	48–188.6	274.5	54.8–856	I vs. II, I vs. HD, II vs. HD
cTnI (ng/dL)	8.3	3.6–16.8	8.2	4.1–13.7	15.9	4.4–30	Ns
Hemoglobin (mmol/L)	11.7	10.3–13	11.0	9.6–12.4	10.1	9.2–11.9	I vs. HD
Sodium (mmol/L)	140.0	138–143	140.0	139–142	140.0	139–141	Ns
Urine osmolality (mOsm/kg H_2_O)	402.0	339–450	319.0	248–399	293.0	239–326	I vs. II, I vs. HD, II vs. HD
Serum osmolality (mOsm/kg H_2_O)	297.0	291–306	304.5	299–317	309.0	296–313	I vs. II, I vs. HD, II vs. HD
Uric acid (mg/dL)	7.5	6.6–8.3	7.4	6.4–8.4	6.7	4.2–8.1	Ns
Serum albumin (g/dL)	3.5	2.8–4	3.5	3.2–4	3.9	3.3–4.2	Ns
C-reactive protein (mg/L)	2.1	1.1–6.4	1.8	0.8–5	5.0	2–9.2	Ns
Procalcitonin (ng/mL)	0.05	0.0–0.1	0.07	0.0–0.1	0.18	0.1–0.3	I vs. HD, II vs. HD
B-lines (LUS)	5.0	3–12	7.0	4–13	8.0	5.5–22	I vs. II, I vs. HD, II vs. HD
ECW (L)	21.1	17.9–24.4	23.3	17.7–26.4	20.7	15.9–28	Ns
ICW (L)	24.9	18–28.3	23.8	20–27.1	22.7	18–26.4	Ns
ECW/ICW	0.9	0.8–1	1.0	0.8–1.1	0.9	0.9–1.1	Ns
Ejection fraction (%)	65.0	60–65	65.0	60–65	60.0	55–65	Ns
GLS	−18.9	−20–−16.7	−18.2	−21.5–−15	−15.9	−19.1–−13.4	Ns
Mitral E/e′	7.0	6.2	8.2	6.4–10	10.0	7.4–11	Ns
LVM index	110.0	100–134	125.0	94–153	139.0	103–187	I vs. HD
VCAM-1 (ng/mL)	1134.5	1005–1415	1300.0	1076–1636	1685.0	1386.5–2240	I vs. II, I vs. HD, II vs. HD
Ang-2 (ng/mL)	2.4	1.9–3.5	2.9	2.2–4.2	5.1	4.2–8.1	I vs. II, I vs. HD, II vs. HD
VEGF-C (ng/mL)	4.7	3.7–6	5.0	4.3–5.9	4.6	3.0–5.6	Ns
Copeptin (pg/mL)	490.0	271–739	572.0	357–960	362.5	322–576	II vs. HD
B-trace protein (ng/mL)	963.0	562.5–1364	1406.0	1014–1711	2088.0	1683–2374	I vs. II, I vs. HD, II vs. HD

6-MWT—6-min walk test; eGFR—estimated glomerular filtration rate; BNP—brain natriuretic peptide; cTnI—cardiac troponin; LUS—lung ultrasonography; ECW—extracellular water; ICW—intracellular water; GLS—global longitudinal strains; Mitral E/e′ ratio—noninvasive measure of left ventricular filling pressure; LVM—left ventricle mass; VCAM-1—vascular cell-adhesion molecule 1; VEGF-C—vascular endothelial growth factor.

## Data Availability

Data are available upon request.

## References

[1] Lv J.-C., Zhang L.-X. (2019). Prevalence and Disease Burden of Chronic Kidney Disease.

[2] Gentile G., Remuzzi G. (2016). Novel Biomarkers for Renal Diseases ? None for the Moment (but One). SLAS Discov. Adv. Sci. Drug Discov..

[3] Augustin H.G., Koh G.Y., Thurston G., Alitalo K. (2009). Control of vascular morphogenesis and homeostasis through the angiopoietin—Tie system. Nat. Rev. Mol. Cell Biol..

[4] Barton W.A., Tzvetkova D., Nikolov D.B. (2005). Structure of the angiopoietin-2 receptor binding domain and identification of surfaces involved in Tie2 recognition. Structure.

[5] Maisonpierre P.C., Suri C., Jones P.F., Bartunkova S., Wiegand S.J., Radziejewski C., Compton D., McClain J., Aldrich T.H., Papadopoulos N. (1997). Angiopoietin-2, a natural antagonist for Tie2 that disrupts in vivo angiogenesis. Science.

[6] Quintanilha J.C.F., Liu Y., Etheridge A.S., Yazdani A., Kindler H.L., Kelly W.K., Nixon A.B., Innocenti F. (2022). Plasma levels of angiopoietin-2, VEGF-A, and VCAM-1 as markers of bevacizumab-induced hypertension: CALGB 80303 and 90401 (Alliance). Angiogenesis.

[7] Yuan H.T., Khankin E.V., Karumanchi S.A., Parikh S.M. (2009). Angiopoietin 2 is a partial agonist/antagonist of Tie2 signaling in the endothelium. Mol. Cell Biol..

[8] Lobov I.B., Brooks P.C., Lang R.A. (2002). Angiopoietin-2 displays VEGF-dependent modulation of capillary structure and endothelial cell survival in vivo. Proc. Natl. Acad. Sci. USA.

[9] Daly C., Pasnikowski E., Burova E., Wong V., Aldrich T.H., Griffiths J., Ioffe E., Daly T.J., Fandl J.P., Papadopoulos N. (2006). Angiopoietin-2 functions as an autocrine protective factor in stressed endothelial cells. Proc. Natl. Acad. Sci. USA.

[10] Ong T., McClintock D.E., Kallet R.H., Ware L.B., Matthay M.A., Liu K.D. (2010). Ratio of angiopoietin-2 to angiopoietin-1 as a predictor of mortality in acute lung injury patients. Crit. Care Med..

[11] Leligdowicz A., Richard-Greenblatt M., Wright J., Crowley V.M., Kain K.C. (2018). Endothelial Activation: The Ang/Tie Axis in Sepsis. Front. Immunol..

[12] Eklund L., Kangas J., Saharinen P. (2017). Angiopoietin-Tie signalling in the cardiovascular and lymphatic systems. Clin. Sci..

[13] Pfister F., Wang Y., Schreiter K., Hagen F.V., Altvater K., Hoffmann S., Deutsch U., Hammes H.-P., Feng Y. (2010). Retinal overexpression of angiopoietin-2 mimics diabetic retinopathy and enhances vascular damages in hyperglycemia. Acta Diabetol..

[14] Akwii R.G., Sajib M.S., Zahra F.T., Mikelis C.M. (2019). Role of Angiopoietin-2 in Vascular Physiology and Pathophysiology. Cells.

[15] Fagiani E., Lorentz P., Kopfstein L., Christofori G. (2011). Angiopoietin-1 and -2 exert antagonistic functions in tumor angiogenesis, yet both induce lymphangiogenesis. Cancer Res..

[16] Chong A.Y., Caine G.J., Freestone B., Blann A.D., Lip G.Y. (2004). Plasma angiopoietin-1, angiopoietin-2, and angiopoietin receptor tie-2 levels in congestive heart failure. J. Am. Coll. Cardiol..

[17] Lim H.S., Lip G.Y., Blann A.D. (2005). Angiopoietin-1 and angiopoietin-2 in diabetes mellitus: Relationship to VEGF, glycaemic control, endothelial damage/dysfunction and atherosclerosis. Atherosclerosis.

[18] Hobohm L., Kölmel S., Niemann C., Kümpers P., Krieg V.J., Bochenek M.L., Lukasz A.H., Reiss Y., Plate K.-H., Liebetrau C. (2021). Role of angiopoietin-2 in venous thrombus resolution and chronic thromboembolic disease. Eur. Respir. J..

[19] Desideri L.F., Traverso C.E., Nicolò M. (2022). The emerging role of the Angiopoietin-Tie pathway as therapeutic target for treating retinal diseases. Expert Opin. Ther. Targets.

[20] Tsai Y.-C., Chiu Y.-W., Kuo H.-T., Lee J.-J., Lee S.-C., Chen T.-H., Lin M.-Y., Hwang S.-J., Kuo M.-C., Hsu Y.-L. (2017). The interaction between fluid status and angiopoietin-2 in adverse renal outcomes of chronic kidney disease. PLoS ONE.

[21] Hennings A., Hannemann A., Rettig R., Dörr M., Nauck M., Völzke H., Lerch M.M., Lieb W., Friedrich N. (2016). Circulating Angiopoietin-2 and Its Soluble Receptor Tie-2 Concentrations Are Related to Renal Function in Two Population-Based Cohorts. PLoS ONE.

[22] Tsai Y.-C., Chiu Y.-W., Tsai J.-C., Kuo H.-T., Lee S.-C., Hung C.-C., Lin M.-Y., Hwang S.-J., Kuo M.-C., Chen H.-C. (2014). Association of angiopoietin-2 with renal outcome in chronic kidney disease. PLoS ONE.

[23] Tsai Y.-C., Lee C.-S., Chiu Y.-W., Lee J.-J., Lee S.-C., Hsu Y.-L., Kuo M.-C. (2018). Angiopoietin-2, Renal Deterioration, Major Adverse Cardiovascular Events and All- Cause Mortality in Patients with Diabetic Nephropathy. Kidney Blood Press. Res..

[24] Tonelli M., Wiebe N., Culleton B., House A., Rabbat C., Fok M., McAlister F., Garg A.X. (2006). Chronic kidney disease and mortality risk: A systematic review. J. Am. Soc. Nephrol..

[25] Lee K.W., Lip G.Y.H., Blann A.D. (2004). Plasma angiopoietin-1, angiopoietin-2, angiopoietin receptor tie-2, and vascular endothelial growth factor levels in acute coronary syndromes. Circulation.

[26] Tsai Y.-C., Lee C.-S., Chiu Y.-W., Kuo H.-T., Lee S.-C., Hwang S.-J., Kuo M.-C., Chen H.-C. (2016). Angiopoietin-2, Angiopoietin-1 and subclinical cardiovascular disease in Chronic Kidney Disease. Sci. Rep..

[27] Tsai Y.-C., Lee C.-S., Chiu Y.-W., Kuo H.-T., Lee S.-C., Hwang S.-J., Kuo M.-C., Chen H.-C. (2015). Angiopoietin-2 as a Prognostic Biomarker of Major Adverse Cardiovascular Events and All- Cause Mortality in Chronic Kidney Disease. PLoS ONE.

[28] Chang F.-C., Liu C.-H., Luo A.-J., Huang T.T.-M., Tsai M.-H., Chen Y.-J., Lai C.-F., Chiang C.-K., Lin T.-H., Chiang W.-C. (2022). Angiopoietin-2 inhibition attenuates kidney fibrosis by hindering chemokine C-C motif ligand 2 expression and apoptosis of endothelial cells. Kidney Int..

[29] Bontekoe J., Lee J., Bansal V., Syed M., Hoppensteadt D., Maia P., Walborn A., Liles J., Brailovsky E., Fareed J. (2018). Biomarker Profiling in Stage 5 Chronic Kidney Disease Identifies the Relationship between Angiopoietin-2 and Atrial Fibrillation. Clin. Appl. Thromb..

[30] Park J.-H., Marwick T.H. (2011). Use and Limitations of E/e’ to Assess Left Ventricular Filling Pressure by Echocardiography. J. Cardiovasc. Ultrasound.

[31] Baber U., Gutierrez O.M., Levitan E.B., Warnock D.G., Farkouh M.E., Tonelli M., Safford M.M., Muntner P. (2013). Risk for recurrent coronary heart disease and all-cause mortality among individuals with chronic kidney disease compared with diabetes mellitus, metabolic syndrome, and cigarette smokers. Am. Heart J..

[32] Kim J.-S., Yang J.-W., Yoo J.S., Choi S.O., Han B.-G. (2017). Association between E/e’ ratio and fluid overload in patients with predialysis chronic kidney disease. PLoS ONE.

[33] Yang F., Wang Q., Zhi G., Zhang L., Huang D., Dangsheng H., Zhang M. (2017). The application of lung ultrasound in acute decompensated heart failure in heart failure with preserved and reduced ejection fraction. Echocardiography.

[34] Lousa I., Reis F., Beirão I., Alves R., Belo L., Santos-Silva A. (2020). New Potential Biomarkers for Chronic Kidney Disease Management-A Review of the Literature. Int. J. Mol. Sci..

[35] Mansour S.G., Bhatraju P.K., Coca S.G., Obeid W., Wilson F.P., Stanaway I.B., Jia Y., Thiessen-Philbrook H., Go A.S., Ikizler T.A. (2022). Angiopoietins as Prognostic Markers for Future Kidney Disease and Heart Failure Events after Acute Kidney Injury. J. Am. Soc. Nephrol..

[36] Levey A.S., Bosch J.P., Lewis J.B., Greene T., Rogers N., Roth D. (1999). A more accurate method to estimate glomerular filtration rate from serum creatinine: A new prediction equation. Modification of Diet in Renal Disease Study Group. Ann. Intern. Med..

[37] Picano E., Frassi F., Agricola E., Gligorova S., Gargani L., Mottola G. (2006). Ultrasound lung comets: A clinically useful sign of extravascular lung water. J. Am. Soc. Echocardiogr..

[38] Mallamaci F., Benedetto F.A., Tripepi R., Rastelli S., Castellino P., Tripepi G., Picano E., Zoccali C. (2010). Detection of pulmonary congestion by chest ultrasound in dialysis patients. JACC Cardiovasc. Imaging.

[39] Picano E., Gargani L., Gheorghiade M. (2010). Why, when, and how to assess pulmonary congestion in heart failure: Pathophysiological, clinical, and methodological implications. Heart Fail. Rev..

[40] Torino C., Gargani L., Sicari R., Letachowicz K., Ekart R., Fliser D., Covic A., Siamopoulos K., Stavroulopoulos A., Massy Z.A. (2016). The agreement between auscultation and lung ultrasound in hemodialysis patients: The LUST study. Clin. J. Am. Soc. Nephrol..

[41] Enright P.L. (2003). The Six-Minute Walk Test. Respir. Care.

